# Cognitive Communication, Voice and Swallowing Difficulties Experienced by Adults With Long‐COVID: A Scoping Review

**DOI:** 10.1111/hex.70595

**Published:** 2026-02-12

**Authors:** Kathleen McTiernan, Caoimhe Hughes, Órla Gilheaney

**Affiliations:** ^1^ School of Linguistic, Speech & Communication Sciences Trinity College Dublin Dublin Ireland; ^2^ Department of Clinical Speech and Language Studies Trinity College Dublin Ireland

**Keywords:** cognitive‐communication, dysphagia, Long‐COVID, post‐COVID‐19, scoping review, voice disorders

## Abstract

**Background:**

Adults with Long‐COVID frequently experience impairments in cognitive‐communication, voice and swallowing, however, few comprehensive reviews of the existing literature has yet to be conducted to map the current research landscape. To go some way toward addressing this gap, this scoping review collected and analysed relevant published studies to identify reported symptoms related to cognitive communication, voice and swallowing in post COVID‐19 patients and the assessments used to identify these difficulties.

**Objective:**

This study aimed to systematically map the existing literature on cognitive‐communication, voice and swallowing difficulties in individuals living with Long‐COVID and the assessments used to identify these difficulties.

**Methods:**

Four databases were searched to identify original research articles aligned with the study's objectives. Studies meeting the inclusion criteria were selected, and the findings were analysed with a specific focus on three key symptom domains: cognitive‐communication, voice and swallowing.

**Results:**

Nineteen studies met the inclusion criteria. A broad range of assessments were used, and a broad range of symptoms were identified related to cognitive‐communication, voice and swallowing difficulties in patients with Long‐COVID‐19. The symptoms reported most frequently in the selected studies included memory deficits, incomplete or inefficient glottic closure, paradoxical vocal fold motion during inspiration, episodes of choking, globus sensation, premature spillage and pyriform sinus residue.

**Conclusion:**

Despite limited prior research in this area, the findings underscore the significant impact that COVID‐19 infection may have on cognitive communication, voice and swallowing functions. Post‐COVID‐19 patients report a wide array of challenges in these domains. As a result, further clinical research is essential to develop patient‐centred care strategies and to equip healthcare professionals with the expertise required for effective management of this group of patients.

## 1 Introduction

Long‐COVID is a condition characterised by systemic symptoms which linger/develop after the acute infection phase of COVID‐19 has passed [1]. These issues are common in those infected with COVID‐19, with over 60% of individuals presenting with one or more complaints for more than 30 days after onset of COVID‐19 [2]. Although robust and in‐depth studies in this domain are limited, frequently reported symptoms include shortness of breath, fatigue, swallowing difficulties, voice problems and cognitive‐communication dysfunction, which negatively impact everyday functioning [1, 3, 4, 5].

To begin, swallowing difficulties have been frequently documented in those living with both COVID‐19 and subsequent Long‐COVID [5, 6]. Research has suggested a range of potential causes of this dysphagia, including: 1) neuromuscular impairments caused by the inflammatory response to the COVID‐19 virus [7]; 2) prolonged ICU admissions and related critical care myopathy [6]; 3) after‐effects of intubation on the airway mechanism [8]; 4) laryngeal hypersensitivity [9]; 5) reflux [9]; 5) COVID‐19 related dyspnoea impairing appropriate breath/swallow coordination [10]; or 6) COVID‐19‐related delirium and fatigue [6, 11], among other sensory factors [12, 13].

Beyond dysphagia, other airway difficulties in those living with Long‐COVID are reported to include voice difficulties, or dysphonia. Postintubation effects are thought to impact significantly on typical voice production in those who have been diagnosed COVID‐19, with prolonged effects beyond the acute phase of illness [10]. Data found in tracheostomized ICU patients who received a fibreoptic endoscopic evaluation of swallowing (FEES) highlighted the prevalence of laryngeal injuries such as oedema, vocal fold palsy, granulomas and mucosal lesions, resulting in COVID‐related dysphonia [6].

This damage to the larynx is likely related to a combined result of the irritation from the endotracheal tube, intubation trauma, as well as disuse atrophy related to intubation [6]. With regards to communication in a broader sense, cognitive‐linguistic difficulties have been reported in those living with Long‐COVID, with hypotheses of causation relating to COVID‐19‐related stroke, mechanical ventilation during hospitalisation, ICU‐related myopathy or prolonged hypoxia occurring as a result of acute respiratory distress syndrome, which is often seen in patients hospitalised with COVID‐19 [14, 15]. Difficulties have been identified primarily in the areas of memory [16], attention, fatigue [3, 17] and executive functioning [17]. These cognitive‐linguistic difficulties have a direct impact on communication, insofar as they often result in impaired verbal fluency and word finding difficulties [16, 18] (e.g., neologisms [18] and paraphasias [17, 19]), in addition to reading, writing and comprehension problems, brain fog [3] and a frequent overall lack of motivation to communicate and impaired health‐related quality of life (QOL) [18].

Evidently, a broad array of post‐COVID‐19 symptoms have been reported that are related to voice, cognitive‐communication and swallowing in those living with Long‐COVID, and early investigations have documented high and upward trending levels of referrals of patients with these Long‐COVID related symptoms to speech and language therapy (SLT) services [9]. However, given the novel nature of such Long‐COVID related difficulties, combined with the limited number of high‐quality studies in this area, and the ever evolving research landscape within this field, SLTs have documented challenges in effectively managing these post‐COVID‐19 symptoms as there is uncertainty regarding aetiology, clinical course and effective means of management [20]. These challenges may result in clinician uncertainty, variable and unstandardised approaches to clinical management, and as such, suboptimal therapeutic outcomes for the patient living with these issues.

As such, to directly address and counteract these concerns, and ultimately improve clinical practice, a comprehensive initial overview of existing recommendations in this field is required. This mapping exercise would contribute to research and our understanding of what knowledge currently exists on this topic, the relative levels the basis of evidence available, and the identified research needs at present. This would act as establishing effective and evidence‐based means of identifying, assessing and managing these Long‐COVID related dysphagia, dysphonia and cognitive‐communication deficits to improve service delivery and ultimately impact patient outcomes and well‐being. Therefore, this scoping review aimed to map and profile the existing literature on cognitive‐communication, voice and swallowing difficulties in individuals living with Long‐COVID and the assessments used to identify these difficulties.

## 2 Methods

### 2.1 Research Design

This scoping review aimed to identify the published literature on the cognitive communication, voice and swallowing difficulties reported in patients with Long‐COVID and the assessments that used to assess these difficulties. A scoping review was selected as the most appropriate design given that this study aimed to provide an initial survey and charting of the breadth and depth of available literature on this previously under‐explored topic. To conduct the review, we followed five of the six steps outlined in the framework developed by Arksey and O'Malley [21] and extended by Levac et al. [22]: (1) Define the research question by clarifying terminology and linking the purpose to the question; (2) Select relevant studies by assessing their applicability and comprehensiveness; (3) Establish inclusion and exclusion criteria to identify studies for review; (4) Chart the data and conduct relevant analysis, as/if appropriate; and 5) Collate, summarise and report the results, including implications for research, policy and practice.

### 2.2 Identifying the Research Questions

The research process began with a comprehensive review of the existing literature on Long‐COVID to gain insight into post‐COVID‐19 symptoms affecting communication and swallowing. This initial literature review played a crucial role in shaping the first draft of the research questions for the scoping review, providing essential background and context for the study. As the review progressed, the research question was refined iteratively, leading to the following final version of the research question: ‘What cognitive‐communication, voice and swallowing difficulties have been reported in adults with Long‐COVID and what assessments were used to identify these challenges?’ For the purpose of this study, the authors conceptualised Long‐COVID in line with the WHO definition [1] as a condition which is characterised by systemic symptoms which linger/develop after the initial acute infection phase of COVID‐19. However, it was noted by authors that as this was an emerging field of research, primary studies may utilise differing definitions to describe the same phenomena.

### 2.3 Identifying Relevant Studies

The search strategy for electronic databases was developed based on the research question and key concepts [21]. With the guidance of a subject librarian, appropriate databases were identified and search strings were refined for optimal results. The final search was conducted on 30 October 2025, across the following databases: PubMed, Embase, CINAHL and Web of Science Core. Additional potentially relevant literature was explored through reference lists from selected articles, as well as searches on Google Scholar. The resulting articles were exported to Covidence, where the second author assessed their relevance to the research question and determined their inclusion in the final data set.

### 2.4 Description of Search Strings and Selection of Articles for Inclusion

Studies directly addressing the research question were selected for inclusion in the data set. Consistent with scoping review guidelines [23] and the study's aim to capture all relevant literature, no exclusion criteria were applied based on publication date or study quality (although all studies were exclusively published post 2020, given the nature and emergence of the COVID‐19 pandemic). The review included primary research from any geographical region, with studies written in English. However, studies involving participants under the age of 18, those with premorbid diagnoses or participants still infected with COVID‐19 were excluded. Due to the broad scope of the research question, systematic and scoping reviews were also excluded. The inclusion and exclusion criteria are detailed in Table 1.

**Table 1:** Inclusion and exclusion criteria for scoping review.

Inclusion criteria	Exclusion criteria
Studies which included people over the age of 18 who reported cognitive communication, voice or swallowing symptoms associated with long‐COVID and the postacute/postinfectious stage of COVID‐19	People with post‐COVID‐19 symptoms who are under the age of 18
Primary research	Patients who had a premorbid diagnosis for example, cancer or myasthenia gravis
Studies from any geographical area	Studies which did not report cognitive communication, voice or swallowing symptoms
Studies conducted within any time period	Studies on individuals who had not yet recovered from COVID‐19
Published journal articles	Studies written in any language except English
	Secondary research‐ systematic/scoping reviews

The initial search strings (Table 2) yielded 37,392 articles on PubMed alone, indicating the need for refinement to better align with the research question. To narrow the focus, the term ‘cognitive dysfunction’ was removed, which significantly improved the precision of the search. The final refined search string resulted in 459 articles across all four databases.

**Table 2:** Initial search string.

Search string
(“Cognitive Dysfunction”[Mesh]) OR ((“Voice”[Title/Abstract] OR “vocal”[Title/Abstract] OR “dysphonia”[Title/Abstract] OR “communication”[Title/Abstract] OR “dysphag*“[Title/Abstract] OR “swallow*“[Title/Abstract] OR (“Voice”[MeSH Terms] OR “Voice Disorders”[MeSH Terms]) OR “Deglutition”[MeSH Terms] OR “Deglutition Disorders”[MeSH Terms] OR “Communication Disorders”[MeSH Terms]) AND (“Post‐Acute COVID‐19 Syndrome”[MeSH Terms] OR (“long covid”[Title/Abstract] OR “post covid”[Title/Abstract] OR “post‐acute covid”[Title/Abstract] OR “long haul covid”[Title/Abstract])))

The refined search strings used for the final search of the databases are displayed in Table 3.

**Table 3:** Refined search string used for final search of databases.

Search string
(“Deglutition Disorders”[Mesh]) OR (“Deglutition”[Mesh])) OR (swallow*[Title/Abstract])) OR (dysphag*[Title/Abstract])) OR (dysphonia[Title/Abstract])) OR (vocal[Title/Abstract])) OR (voice[Title/Abstract])) OR (“Voice”[Mesh])) OR (“Voice Disorders”[Mesh])) OR (“Communication Disorders”[Mesh])) OR (cognitive communication[Title/Abstract])) AND (((((“Post‐Acute COVID‐19 Syndrome”[Mesh]) OR (long covid[Title/Abstract])) OR (post covid[Title/Abstract])) OR (post‐acute covid[Title/Abstract])) OR (long haul covid[Title/Abstract]))

**Figure 1:**
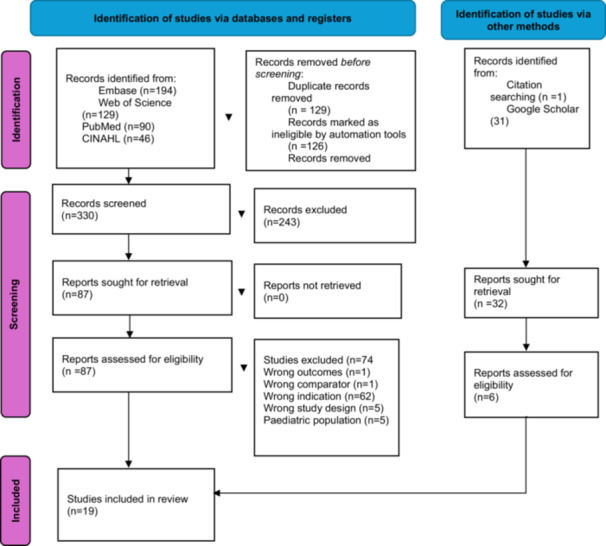
PRISMA flow diagram displaying article selection process.

A PRISMA flow diagram [24] was utilised to document each stage of the article selection process, tracking the number of articles screened and those ultimately selected for inclusion in the data set (Figure 1).

Articles were imported into Covidence for screening and review [25]. First, duplicate articles were removed (*n* = 129) after an initial screening of titles. The abstracts of the remaining articles were then reviewed to ensure relevance to the research question, leading to the removal of articles that were only tangentially related (*n* = 243). Full texts of the remaining articles were thoroughly reviewed, resulting in the exclusion of 74 articles that did not meet the criteria. Six additional articles were included through citation and Google Scholar searching. The first author reviewed the final selection, confirming agreement with the chosen studies. Ultimately, 19 articles directly related to the research question were included in the final data set.

### 2.5 Charting the Data

The selected articles were organised in an Excel spreadsheet for data charting. A data extraction chart was exported from Covidence [25] and modified to fit the study's needs. The following headings were included as areas to extract key findings on: study title, author(s), year of publication, language, study location, study design, study aim, methodology, participant recruitment method, number of participants, participant demographics, study focus, illness severity/hospitalisation status, assessments used and key conclusions of the primary studies.

### 2.6 Collating, Summarising and Reporting the Results

To facilitate analysis of findings from primary studies, the details of eligible and included articles were then organised and summarised within an Excel Sheet. To ensure consistency in reporting, a standardised template for data charting was created, enabling clear identification of conflicting evidence and research gaps, which could inform future studies [21]. An overview of the included studies was constructed by reviewing the initial data, followed by a descriptive summary of the volume, characteristics and distribution of the evidence found within primary studies. The results were extracted from primary studies and narratively and descriptively presented under the three key headings, which were derived from symptoms detailed within the research question: cognitive communication, voice and dysphagia. Tables and figures were generated to visually summarise the key findings, which were extracted from the selected articles.

## 3 Results

### 3.1 Overview of the Characteristics of Selected Studies

A total of nineteen studies were included in the scoping review, all published in English between 2020 and 2025. Among these, seven studies were conducted in the United States, while the remaining studies originated from Belgium (*n* = 1), India (*n* = 1), Italy (*n* = 1), Ireland (*n* = 3), Lebanon (*n* = 1), Poland (*n* = 1), Spain (*n* = 1), the United Kingdom (*n* = 1), Brazil (*n* = 1) and China (*n* = 1). In terms of study design, six studies (32%) utilised a cohort approach, four (21%) employed clinical case studies, three (16%) followed a cross‐sectional design, two studies (11%) used a survey design, one study (5%) used a case series format, one study (5%) implemented an experimental design, one study (5%) was an outcomes study and one (5%) study utilised a phenomenological qualitative design. The selected studies recruited participants from the following care settings: primary care (16%, *n* = 3), secondary care (31%, *n* = 6), specialty care (21%, *n* = 4), both primary and secondary care settings (16%, *n* = 3) and community settings (16%, *n* = 3).

### 3.2 Overview of Participant Demographics, Assessments and Symptoms

**Figure 2:**
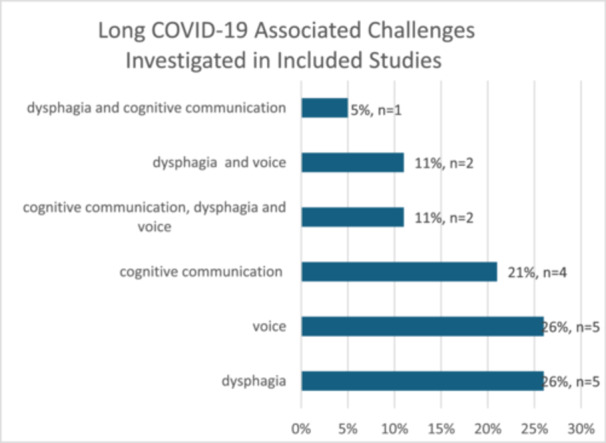
Long‐COVID‐19 associated challenges investigated in included studies.

All of the selected studies included participant demographics, descriptions of the assessments used, and details about patients' symptoms. In total, there were 1602 participants involved across the 19 studies and the average age of participants was 49 years, with an age range of 18 to 91. The studies explored patient challenges with voice, swallowing and cognitive communication difficulties, as well as various combinations of these challenges related to Long‐COVID‐19 (Figure 2).

Most of the studies focused on swallowing difficulties (52.63%, *n* = 10) or voice difficulties (47.36%, *n* = 9), while seven studies (36.84%) investigated cognitive communication challenges and a small cohort (26.31%, *n* = 5) explored more than one symptom experience.

### 3.3 Overview of Cognitive Communication Studies

Three of the seven studies (1, 3, 18) investigating cognitive‐communication challenges utilised a combination of adapted standardised assessments to evaluate various cognitive domains (e.g., attention, executive functioning, memory, language functioning/fluency, orientation and visuospatial skills). In one study (1), general cognitive‐communication abilities were assessed using the Montreal cognitive assessment (MOCA) and mini mental state exam (MMSE) assessments, supplemented by subtests from other assessments. Five studies (1–3, 16–17) also included self‐reported cognitive‐communication symptoms. Processing speed was evaluated through subtests from standardized assessments, for example: the WAIS‐IV coding subtest (91), and via a computer‐based task (2).

Word‐finding difficulties were noted in six studies (1, 2, 15, 16, 17, 18), while memory challenges, including working memory issues, were reported across all studies. Slowed processing and response times were observed in two studies (1, 2), and difficulties with organisation, a key aspect of executive functioning, were highlighted in studies 1 and 3. Cognitive fatigue was reported in several studies (2, 15, 17, 18). Participants experiencing ‘brain fog’ scored higher on depression and posttraumatic stress assessments (2), and one study (3) noted the onset of new anxiety and depressive symptoms following COVID‐19. Two of the three studies examining cognitive‐communication difficulties reported that participants had poor quality‐of‐life outcomes (2, 3).

### 3.4 Overview of Voice Studies

Studies focusing on voice (4, 5, 6, 7, 8, 9, 10, 15, 17) aimed to both describe the voice symptoms reported by patients and evaluate the management of these symptoms (8, 10). Several studies (4, 5, 15, 17) examined patients with COVID‐19 which varied in severity, while the remaining studies focused on patients who were hospitalised with the infection. One study (6) compared symptom severity between hospitalised and nonhospitalised patients. Most studies involved patients who were not intubated during their COVID‐19 illness (4, 5, 8, 9, 17). Three studies (6, 10, 15) included patients who had been intubated and nonintubated and discussed differences in presentation between these groups.

The voice studies employed a wide range of assessments to evaluate voice difficulties. Stroboscopy was utilised in several studies (4, 5, 7, 8, 10) to observe and describe vocal fold movements. One study (9) conducted a biomechanical analysis of a voice recording to detail voice symptomatology, using an iPhone app to analyze 22 parameters describing overall biomechanics and the biomechanical wave. Voice quality ratings were performed by a clinician using the measuring grade, rougness, breathiness, asthenia, and strain (GRBAS) scale (study 10) and maximum phonation time (MPT) (study 5). To assess the impact of voice difficulties on patients' QOL, various scales were employed, including the voice handicap index (VHI), Vocal cord dysfunction questionnaire, and voice‐related quality of life (VRQOL) assessments (studies 4, 6, 7, 8, 9, 10). Additionally, patients in two studies (8, 13) underwent an ENT examination.

A wide range of symptoms were reported across the voice studies, with varying degrees of severity. Mild to moderate symptoms were noted in three studies (6, 9, 10), while severe symptoms were observed in four studies (4, 5, 7, 8), while other studies did not specify severity profiles (15, 17). Patients across studies commonly reported aphonia, hoarseness, loss of vocal stamina, strain and dry throat or larynx. The median GRBAS score reported was G2R1B0A1S1, indicating a moderate voice abnormality in one study (10). In one clinical case study, a patient demonstrated a MPT of just 3 s, placing them below average (5). Stroboscopy revealed abnormal mucosal waves (8, 10), incomplete or inefficient glottic closure (8, 10), unilateral vocal cord paresis (10), muscle tension dysphonia, intra‐arytenoid oedema, suspected laryngo‐pharyngeal reflux and vocal cord palsy postextubation (15).

One study (9) found that women showed changes in fundamental frequency, an imbalance in vocal fold structure, increased glottic closing force and glottic insufficiency, while men did not exhibit such changes in frequency or glottic closing force during biomechanical analysis. Other findings included decreased vocal amplitude (10), structural imbalances or etiologies (9, 10) and throat clearing and coughing (8, 17). Additionally, one study reported reflux, laryngeal hypersensitivity in nonintubated patients, and laryngeal stenosis in those who had been intubated (study 10). Vocal fatigue was prevalent among 10.35% of patients with severe dysphonia (8).

Inappropriate adduction of the vocal cords during inspiration was reported in four studies (4, 5, 7, 15) and was diagnosed as paradoxical vocal fold movement disorder in two of these (5, 7). Patients with this condition did not exhibit signs of reflux (4, 5, 7). One clinical case study described a patient's voice as severely impaired, characterised by low volume and a monotonous tone, with a notable absence of mucosa on the vocal cords (7). Voice symptoms were more pronounced in patients who had been hospitalised compared to those who were not hospitalised (6). Intubated patients sought laryngology services sooner than nonintubated patients and required more extensive care (10). Impact scale scores ranged from mild to severe across studies (4, 6, 7, 8, 9, 10), with VHI scores indicating varying levels of disability (6, 7, 8, 9, 10). In one study, 18 (86%) of the 21 patients had mild disability based on VHI results (9), while other studies reported that patients had moderate to severe disability scores (7, 8). VHI scores were higher in patients with dysphonia compared to those without voice difficulties (10) and were also elevated in patients who had been critically ill during the acute phase of COVID‐19 (6). Patients with postintubation laryngotracheal stenosis following COVID‐19 had higher scores across all quality‐of‐life assessments, indicating a significantly reduced QOL (10).

### 3.5 Overview of Dysphagia Studies

The studies investigating dysphagia (6, 10, 11, 12, 13, 14, 15, 16, 17, 19) aimed to assess the prevalence and characteristics of dysphagia in patients recovering from COVID‐19. Studies investigating participants with dysphagia were represented by a mix of individuals who had/had not been hospitalised due to COVID‐19, and the studies included both intubated and nonintubated patients. Notably, two studies (12 and 14) found that the majority of patients with dysphagia had been intubated, while most studies (6, 11, 13, 15, 16, 17, 19) reported a smaller proportion of patients had been intubated. The studies focusing on dysphagia (6, 10, 11, 12, 13, 14, 15, 16, 17 and 19) primarily reported mild or subclinical symptoms. One study (6) found that the severity of dysphagia was greater in patients that had been intubated, with severity levels increasing for each day of intubation. Additionally, in another study (12) a strong correlation was reported between intubation and a penetration and aspiration scale (PAS) score of 7 or 8, indicating aspiration. This study also noted that dysphagia symptoms were prevalent among the majority of tracheostomized patients. Notably, dysphagia symptoms were also identified in nonintubated patients (11).

Among the challenges identified through instrumental swallowing assessments (11, 12, 13, 14, 15), the most common issues included premature spillage and pharyngeal residue (both reported in studies 12 and 13), as well as reduced hyolaryngeal movement, pharyngeal swallow trigger delay, reduced tongue base retraction, reduced pharyngoesophageal segment opening and diminished soft palate elevation, which were reported in study 12. The findings from the Mann assessment of swallowing ability (MASA) assessment in one study (14) indicated that less than one third of patients were at risk of aspiration, while another study (12) reported that very few patients met the clinical criteria for nothing by mouth (nil per os).

One study (13) utilising the FEES examination found that seven (17%) of the 41 participants experienced inadequate velopharyngeal closure, along with instances of penetration and aspiration.

In most studies utilising the Functional Oral Intake Scale (FOIS) assessment (13, 14), overall scores were 6 or lower, suggesting the presence of swallowing difficulties, although this trend was reversed in one study (19). Qualitative findings in several studies (e.g., 11, 13, 17), supported these findings, with participants reporting additional experiences of ‘difficulty swallowing’, ‘choking episodes’, ‘lump in the throat’ and ‘nasal regurgitation’.

Furthermore, results from the swallowing quality of life (SwalQOL) assessment in study 11 revealed that four of the eight participants reported a poor health‐related QOL due to their swallowing difficulties, which impacted their ‘timing of meals’, ‘sleep’ and ‘desire to eat’.

### 3.6 Summary of Results

A wide range of difficulties related to cognitive communication, voice and swallowing were reported across the 19 studies included in this scoping review. The cognitive communication studies primarily identified neurocognitive impairments, with common issues including memory difficulties, processing challenges and deficits in attention and working memory. Findings from the voice studies suggest that respiratory complications associated with COVID‐19 may lead to significant laryngeal dysfunction, adversely affecting voice quality and respiratory mechanics. The dysphagia studies underscored the prevalence and severity of swallowing difficulties in post‐COVID‐19 patients, revealing that hospitalised participants, particularly those who were intubated or tracheostomized, experienced more severe symptoms than their nonhospitalised counterparts. Specific assessments, such as the MASA and FOIS, indicated varying degrees of aspiration risk and highlighted the impact of swallowing difficulties on QOL.

## 4 Discussion

This scoping review demonstrated that cognitive communication, voice and swallowing difficulties are prevalent amongst those living with post‐COVID‐19 sequelae and those with Long‐COVID, as documented in the available literature and the assessments used to identify these difficulties. However, research designs and locations within primary studies were heterogeneous, with a range of reported issues investigated using a myriad of tools with varying psychometric properties, thus limiting applicability and generalisability of findings and necessitating future rigorous research in this field to improve patient‐centred care.

The eligible literature reported that dysphagia and dysphonia are the most common difficulties reported to SLT services by those living with Long‐COVID [20]. A broad range of dysphagia presentations were reported in included studies, with frequent subjective reports of choking [26, 27] and globus sensation [27, 28]. The most common findings on instrumental analysis within primary studies included premature spillage and residue in the pyriform sinus [27, 29]. With regards to voice, glottic insufficiency and reduced vocal fold adduction were reported most commonly here [28, 30, 32], with other frequent difficulties including changes to the mucosal wave [28, 33] and structural aetiologies [28, 34]. It is hypothesised that COVID‐19 related inflammation, neurological concerns, medical interventions and/or psycho‐emotional factors may contribute to both the vocal and swallowing deficits documented here, although causation cannot currently be established due to a lack of rigorous and appropriate available studies. Furthermore, these tentative results should be interpreted with caution and readers are advised to avoid over‐generalisations regarding potential prevalence rates, given the myriad of study design and rigour concerns identified here. However, with this cautious approach in mind, it appears that dysphagia and dysphonia are areas requiring further investigation in order to establish the true extent and impact of these concerns and to therefore improve the delivery of patient‐centred care for this cohort Table 4.

**Table 4:** Overview of selected studies.

Study	Design/aims/sample	Primary/secondary/specialty	Assessment	Results	Cognitive	Voice	Dysphagia
Study 1: Hellmuth et al. [35]. Persistent COVID‐19‐ associated neurocognitive symptoms in nonhospitalised patients	Design: Clinical case study Aims: To discuss persistent neurocognitive impairments COVID‐19 in patients who had mild disease symptoms Sample: *n* = 2	Specialty	Self‐report. Global cognition‐ Montreal cognitive assessment (MOCA). Mini mental state exam (MMSE) Memory‐California Verbal Learning Test‐3 (16‐word) Rey‐Osterreith Complex Figure Attention/working memory‐WAIS‐IV Digit Span. Forward span & Backward span Fluency‐ D‐KEFS Letter Fluency. D‐KEFS Category Fluency. D‐KEFS Design Fluency Processing speed‐WAIS‐IV Coding. d‐KEFS Trail Making. D‐KEFS Colour‐Word Interference Visuospatial‐ NAB Visual Discrimination. Rey‐Osterreith Complex Figure Copy	Patient 1‐Memory difficulties, processing difficulties, error prone task execution Patient 2‐Word finding difficulties. Both patients‐difficulties organising, working memory, average attention, letter and category fluency.	✓	✕	✕
Study 2: Jennings et al. [36]. Comprehensive clinical characterisation of brain fog in adults reporting Long‐COVID symptoms	Design: Cross‐sectional observation Aims: To provide a clinical characterisation of self‐reported brain fog in adults with Long‐COVID symptoms Sample: *n* = 108	Secondary	Self‐report. Computer assisted cognitive tasks‐ SRT and CRT tasks	Memory, word finding difficulties, fatigue, slower response times	✓	✕	✕
Study 3: Whiteside et al. [37]. Neurocognitive deficits in severe COVID‐19 infection: Case series and proposed model	Design: Clinical Case Series Aims: To address cognition in post‐COVID‐19 patients by describing patients in acute rehabilitation to create a model of cognitive difficulties post‐COVID‐19 Sample: *n* = 3	Primary	Self‐reported. Orientation‐asked to provide details of themselves. Name 6 most recent presidents of USA Attention/working memory‐WAIS‐IV Digit Span, TSAT, Oral Trail Making Test, Part A Verbal Memory and Learning‐Hopkins verbal Learning Test Revised and Story Memory from RBANS Update Language functioning‐ Receptive‐Complex Ideational Material. Expressive‐WAIS‐IV Similarities and Vocabulary subtests (expressive) Verbal Fluency (Letter fluency (FAS)and Animal Fluency Executive Functioning‐ Oral Trail Making Test, Part B Verbal Fluency (Letter fluency [FAS] and Animal Fluency), TSAT. Independent living Scales‐health and safety classification. Psychological Functioning‐Beck Anxiety Inventory, Geriatric Depression	Patient 1‐Expressive language‐below average, receptive language within normal limits. Variable attention and executive functioning across tasks. Significant anxiety Patient 2‐ Language function variable, executive functioning difficulties, variable attention. Anxiety and depression Patient 3‐impaired verbal fluency and receptive language, executive functioning, attention within normal limits. No symptoms of emotional distress All patients‐significant difficulty in encoding of new verbal information (less structured memory tasks	✓	✕	✕
Study 4: Garg et al. [31]. Post‐COVID‐19 vocal cord dysfunction	Design: Clinical case study Aims: To describe vocal symptoms in post‐COVID clinic case Sample: *n* = 1	Secondary	Flexible video‐endoscopy, Vocal Cord Dysfunction questionnaire	Almost complete adduction of vocal folds and pharyngeal wall contraction on inspiration, normal abduction at rest	✕	✓	✕
Study 5: Lechien et al. [32]. Post‐COVID‐19 paradoxical vocal fold movement disorder	Design: Clinical case study Aims: To describe voice symptoms in a patient presenting to their clinic Sample: *n* = 1	Secondary	Maximum phonation time (MPT), Videolaryngostroboscopy	Paradoxical vocal fold movement disorder. Inspiratory and expiratory paradoxical movement of the vocal folds on inspiration and expiration	✕	✓	✕
Study 6: Bouldin et al. [38]. Otolaryngologic symptom severity post SARS‐CoV‐2 infection	Design: Survey design Aims: To assess laryngological symptoms in patients post severe COVID‐19 infection and to determine if there is a correlation between symptom severity and disease severity Assess voice/swallowing symptoms and severity correlation Sample: *n* = 470	Primary/ secondary	Voice Handicap Index‐10 (VHI‐10) Dyspnoea Index (DI) Cough Severity Index (CSI) Eating Assessment Tool‐10 (EAT‐10)	Eat‐10: Hospitalised‐ mean score 3.6 (abnormal), nonhospitalised mean: 1.7 (within normal limits), Over 12 months post infection: Hospitalised‐ mean: 2.9 (within normal limits), nonhospitalised‐ mean: 0.2 VHI‐10 scores were statistically significantly greater in hospitalised compared to nonhospitalized participants The DI was significantly greater in the hospitalised participants than nonhospitalized. CSI mean scores to be significantly elevated in those who were hospitalised compared to nonhospitalized. Dysphagia symptoms captured by EAT‐10 score were significantly greater in hospitalised versus nonhospitalized participants.	✕	✓	✓
Study 7: El Kik et al. [30]. Post‐COVID‐19 paradoxical vocal cord movement and dysfunctional dysphonia: a clinical case	Design: Clinical case study Aims: To describe a case of a post‐COVID‐19 patient with both paradoxical vocal fold movement and dysfunctional dysphonia post COVID‐19 infection Sample: *n* = 1	Primary	The Voice Handicap Index 10 (VHI‐10) chest computed tomography (CT), Bronchoscopy	Paradoxical vocal fold movement and type 4 dysfunctional dysphonia of the Koufman's classification. Severe dysphonia. Inaudible, low and monotonous voice. Bronchoscopy: Adduction of vocal folds on inspiration. VHI: 31/38	✕	✓	✕
Study 8: Jeleniewska et al. [33]. Isolated severe dysphonia as a presentation of post‐COVID‐19 syndrome	Design: Cohort study Aims: To assess the clinical management of severe, isolated dysphonia during post‐COVID‐19 syndrome. Sample: *n* = 158	Specialty	Pre and postexamination protocol consisted of subjective voice self‐assessment and routine laryngological examination, followed by an instrumental examination by means of Laryngovideostroboscopy (LVS) and High‐Speed Videolaryngoscopy (HSV)	Pretreatment: Moderate/severe dysphonia, mucosal atrophy, incomplete closure Moderate/severe voice symptoms. Aphonia, hoarseness with vocal fatigue and loss of voice strain and stamina. Dry throat and larynx, frequent throat clearing and cough VHI‐mean: 62. VRQOL‐mean: 57. ENT‐subacute pharyngolaryngitis with chronic erythema, chronic lesions, dry mucosa, red, dry, stiff vocal folds, dilated vessels. Mucosal and glandular atrophy associated with thick ‘frothy’ mucus on the vocal folds in some patients. Videostroboscopy: Deviations in the similarity and regularity of vocal fold vibrations, incomplete glottic closure and absence of mucosal wave. Treatment: Treatment included short‐term systemic steroids in decreasing doses, moisturising inhalations with hyaluronic acid, and protective agents against Laryngopharyngeal Reflux Posttreatment: Improvement of the structural and functional state of the larynx was observed posttreatment. Posttreatment improvement in vocal function and voice quality in all the examined patients	✕	✓	✕
Study 9: Romero Arias and Betancort Montesinos [34]. Voice Sequelae following recovery from COVID‐19	Design: Experimental design Aims: To identify the patterns of behaviour in the biomechanical correlates of people post‐COVID‐19 with sequelae in voice Sample: *n* = 21	Secondary	VHI, Biomechanical analysis	VHI‐86% presented with mild disability. Mean: women‐11.18, men‐11.2. The VHI‐30 in this sample implies differences in voice perception between the sexes. At the biomechanical level, a COVID‐19 infection affects in the same way as other infectious processes.	✕	✓	✕
Study 10: Shah et al. [28]. Long‐term laryngological sequelae and patient‐reported outcomes after COVID‐19 infection	Design: Cross sectional design Aims: To examine prevalence, characteristics, quality of life (QOL) assessments, and long‐term effects of interventions for laryngeal dysfunction after recovery from COVID‐19 infection Sample: *n* = 57	Primary/ secondary	Stroboscopy, EAT‐10, Patient‐reported QOL indices were Dyspnoea Index (DI), Cough Severity Index (CSI), Voice Handicap Index‐10 (VHI‐10), Eating Assessment Tool‐10 (EAT‐10) and Reflux Symptom Index (RSI)	Dysphagia diagnosis‐25% of patients, 17.5% were diagnosed with Laryngotracheal stenosis (LTS) (all intubated patients), 15.8% presented with laryngopharyngeal reflux (LPR), 8.8% presented with globus, laryngeal hypersensitivity found in 22.8% of patients (majority were not intubated). Median baseline EAT‐10‐4/40 (abnormal). Dysphonia diagnosed in 70% patients, dysphagia in 25% patients, COVID‐related laryngeal hypersensitivity in 23%, and laryngotracheal stenosis (LTS) in 18% patients. Of the 17 patients who underwent voice therapy, 11 (64.7%) reported improvement in their symptoms and 2 (11.8%) patients reported resolution. VHI scores decreased for patients who reported symptom improvement. 70% patients with LTS required > 1 procedural intervention before symptom improvement. Improvement across QOL indices was seen in patients with LTS.	✕	✓	✓
Study 11: Marchese et al. [26]. Oropharyngeal dysphagia after hospitalisation for COVID‐19 disease: Our screening results	Design: Cohort study Aims: To describe the prevalence, severity and features of OPD after hospitalisation and recovery from the COVID‐19 disease Sample: *n* = 117	Specialty	EAT‐10, GUSS, FEES, SwalQOL	Prevalence of upper dysphagia after hospitalisation for SARS‐CoV‐2 is not anecdotal and that probably this long‐lasting sequela has a psychogenic etiology. 50% abnormal health related QOL in oropharyngeal dysphagia with a mean Swal‐QoL score of 69.73. The most affected domain was the ‘time of meals’ (mean score 65) following by the ‘sleep’ (mean score 66) and ‘eating desire’ (mean score 72). 1/8 cases showed increased risk for aspiration and did not show endoscopic signs of oropharyngeal dysphagia.	✕	✕	✓
Study 12: Rajski et al. [29]. Dysphagia outcomes in COVID‐19 patients: experiences in long‐term acute care hospital (LTACH)	Design: Outcomes research Aims: To describe this unique dysphagia management experience to improve future patient care in LTACH patients with respiratory failure Sample: *n* = 213	Speciality	Demographic information, videofluoroscopic swallow study (VFSS) reports with Penetration and Aspiration Scale (PAS) scores and SLP notes were reviewed	There was a strong association (*p* = 0.001) between patients who had tracheostomy placed within 33 days of VFSS and recommendation for thin liquids. Upon discharge, the majority of patients (83.57%) transitioned successfully to oral diets, however, a strong association (*p* = 0.009) between higher age (≥ 62) and nothing by mouth (NPO) at discharge was demonstrated	✕	✕	✓
Study 13: Sharma et al. [27]. Dysphagia in post‐COVID‐19 patients—a prospective cohort study	Design: Cohort study Aims: To investigate dysphagia presentation, Functional Oral Intake Scale (FOIS) results and findings in Fiberoptic Endoscopic Evaluation of Swallowing (FEES) in patients reporting swallowing difficulties, post recovery from COVID‐19 Sample: *n* = 41	Primary/secondary	FOIS, FEES, ENT	19 patients presented with difficulty in swallowing, 11 reported choking episodes, 5 reported a lump in throat after swallowing and 6 patients reported nasal regurgitation FOIS‐ Level 1 in 8 patients, Level 2–3 in 12 patients, Level 4–6 in 11 patients and Level 7 in 10 patients. FEES‐ Inadequate velopharyngeal closure indicating palatal palsy (7 patients) Premature spillage (3 patients)	✕	✕	✓
Study 14: Webler et al. [39]. Dysphagia characteristics of patients post SARS‐CoV‐2 during inpatient rehabilitation	Design: Cohort study Aims: To investigate dysphagia in patients recovering from SARS‐CoV‐2 admitted to acute inpatient rehabilitation by summarising clinical swallow evaluation and videofluoroscopic swallow study findings Sample: *n* = 40	Primary	VFSS, MASA, FOIS	93% had FOIS score of 6 or less 20% were NPO 8% could consume nutrition orally without any modifications 62% had consistency modifications for oral nutrition 10% had oral diet and supplementation through a gastrostomy tube MASA‐45% unlikely to aspirate VFSS‐30% demonstrated a PAS score of 6 or greater 25% showed penetration without ejection	✕	✕	✓
Article 15 Chalmers et al. [9]. A retrospective study of patients presenting with speech And language therapy needs within multidisciplinary Long‐COVID services: A service evaluation describing and Comparing two cohorts across two NHS Trusts	Design: Retrospective Cohort study, describing two cohort case series Aims: What is the typical presentation of patients referred into MDT Long‐COVID services with SLT needs? Sample: Cohort 1 Bolton *n* = 82 Cohort 2 East Suffolk *n* = 86	Secondary	Reason for referral made to SLT Services SLT diagnosis	Referral reasons: Dysphonia: Bolton: 73.2% (60/82) East Suffolk: 64.0% (55/86) Dysphagia: Bolton: 34.2%(28/82) East Suffolk: 58.1% (50/86) Cognitive communication difficulties: Bolton: (15.9%,13/82) East Suffolk: 61.6% (53/86) SLT diagnosis: Bolton service: Dysphonia: 57.7% (45/78) Dysphagia: 30.8% (24/78) Cognitive communication difficulties: 14.1% (11/78) East Suffolk: Dysphonia: 48.7% Dysphagia: 29.0%(22/76) Cognitive communication difficulties: 52.6% (40/76)	✓	✓	✓
Study 16 Gilheaney et al. [40]. The prevalence and nature of communication and swallowing difficulties among adults with long‐COVID	Design: Survey Aims: To investigate the prevalence, nature and severity of communication and swallowing difficulties in adults with Long‐COVID. Sample: *n* = 79	Primary	16‐item anonymous survey regarding the prevalence, nature and severity of communication and swallowing difficulties associated with Long‐COVID	96% (*n* = 76) reported cognitive communication difficulties, severity: moderate 73% (*n* = 58) reported swallowing difficulties, severity: moderate	✓	✕	✓
Study 17 Gilheaney et al. [41]. Exploring the lived experiences and perspectives of individuals with communication and swallowing difficulties associated with Long‐COVID	Design: Phenomenological qualitative study Aims: To establish an understanding of the presence, severity and trajectory of swallowing and communication difficulties as a symptom of Long‐COVID among adults. To investigate the psychosocial impact of these characteristics and explore supports and barriers to recovery Sample: *n* = 7	Primary	Semi‐structured interviews	Theme: Dysphagia‐related issues ‐Modifications to diet and mealtime ‐Oral phase difficulties ‐Pharyngeal phase difficulties ‐Oesophageal phase difficulties Theme: Communication‐related issues ‐Receptive language difficulties ‐Expressive language difficulties ‐Voice difficulties ‐Speech fluency difficulties Psychosocial impact of dysphagia and communication disorders ‐Family and home life ‐Social life ‐Professional life ‐Mental health Supports for swallowing difficulties: ‐prompting to eat slowly, ‐preparing suitably modified meals ‐being patient Supports for communication ‐being understanding of communication abilities, ‐recognising difficulties ‐prompting or helping Barriers to recovery: ‐limited access to specialised care for both swallowing and communication difficulties	✓	✓	✓
Study 18 Cummings [16]. Cognitive‐linguistic difficulties in adults with Long‐ COVID: A follow‐up study	Design: Follow up cohort study Aims: To examine the cognitive‐linguistic skills of 41 adults with Long‐COVID at two time points 6 months apart and compare results Sample: *n* = 41	Primary	Test 1: Immediate recall Test 2: Picture description Test 3: Sentence generation Test 4: Narration Test 5: Phonemic (letter) fluency Test 6: Semantic (fluency 1 Test 7: Semantic fluency 2 Test 8: Narration Test 9: Procedural discourse 1 Test 10: Procedural discourse 2 Test 11: Confrontation naming Test 12: Delayed recall	Difficulties in immediate and delayed verbal recall persist long after the onset of COVID symptoms Improvements were noted in verbal fluency and the informativeness of spoken discourse	✓	✕	✕
Study 19 César et al. [42]. Assessment of smell and swallowing in patients with post‐COVID‐19 syndrome	Design: Observational fross‐sectional Aims: To evaluate the olfactory and swallowing aspects in patients with post‐COVID‐19 syndrome Sample: *n* = 62	Primary	Olfactometry: Connecticut test Lip and tongue pressure: PLL equipment from Pró‐Fono® Swallowing: assessment using foods with three different consistencies, the FOIS and the ASHA‐NOMS	Altered sense of smell 83.71% of the sample, impaired swallowing in 16.13% of cases, majority presented functional swallowing Average pressure values: for the lips 45.86 (± 19.93) kPa, for the apex of the tongue 31.93 (± 18.45) kPa, and for the dorsum of the tongue 32.28 (± 17.66) kPa	✕	✕	✓

The most common cognitive communication difficulties reported within the limited number of eligible studies here were issues with memory and organisation, delayed processing and word finding difficulties [16, 17, 18], yet reporting rates varied significantly, and this combined with the scoping nature of this study hindered analysis at a deeper level. Furthermore, it is hypothesised that although cognitive‐communication difficulties were reported at a lower rate than other concerns within included records here, they may be more commonly experienced than has yet been documented, with potential masking of subtle higher level issues and over‐prioritisation of physical signs of swallowing or voice concerns [18]. However, again, one must interpret these early results with caution and use the findings primarily as an impetus for future, more systematic research into this area to promote positive clinical developments and ultimately improved patient outcomes.

As discussed above, a unifying finding across primary studies was significant variability in reporting rates. It is hypothesised here that a high level of heterogeneity in patient demographics, definition of Long‐COVID, time since onset of COVID‐19 infection, severity of experienced symptoms and variability in assessment protocols are core contributors to this variability. For example, significant gender disparities in recruited participants were noted, with increased female representation in studies focusing on cognitive communication and voice difficulties [34]. Although this aligns with literature reporting voice issues to occur more commonly in females [43], it may introduce an element of confounding in results, which limits our understanding of the full patient experience. Additionally, all included studies which focused on dysphagia recruited hospitalised patients, in recognition perhaps of the role of intubation and severe respiratory and/or neurological concerns in the development of dysphagia [38]. However, this may limit our understanding of individuals who did not require acute medical care but may still develop postviral swallowing difficulties.

Furthermore, all included articles reporting dysphagia symptoms recruited patients over the age of 50 years of age. Although the risk of developing swallowing difficulties in general increases in those aged 50 years old and above [44], and there is early emerging research suggesting a correlation between higher age of contracting COVID‐19 and an nothing by mouth (NPO) status at discharge [29], this restricts our available knowledge regarding the range of dysphagia symptoms experienced across the full lifespan. As such, future research which broadens previous criteria across demographics, illness trajectory and severity and data collection techniques is necessitated in order to deepen and solidify our understanding of these symptoms and their impact.

### 4.1 Review Limitations

Limiting factors within the eligible literature have been discussed above, yet some crucial internal factors must also be considered. For example, strict eligibility criteria excluded those with previous diagnoses which may have caused premorbid swallowing or communication deficits. Although this reduces detection bias, it may limit our understanding of the potential for Long‐COVID to impact/exacerbate pre‐existing issues. Therefore, this observation may limit practical application of results in a clinical sense currently, as clinicians cannot reliably generalise results of primary studies to their own clinical caseloads, thus potentially hindering current service delivery, with potential impact on patient outcomes and well‐being. Additionally, searches were relatively narrow and only identified articles published in English, secondary to time and translation resource restrictions, thus potentially excluding crucial findings. As such, future research would benefit from further consideration of search methods and eligibility criteria, with added potential benefit from inclusion of critical analysis of study quality in the process of completing a systematic review on this topic. Finally, a limitation within included primary studies was the lack of explicit PPIE, which potentially restricts our understanding of the full spectrum of the long‐COVID experience. It is recommended that future research in this area utiliszing the ACTIVE framework [45] is conducted in order to advocate for and champion PPIE within future evidence‐based investigations on this topic.

## 5 Conclusion

This review provided an initial synopsis of the available literature which discusses cognitive communication, voice and swallowing difficulties amongst those living with Long‐COVID, suggesting that there may be a broad range of such concerns reported by this cohort. This initial mapping exercise suggests that there may be a need for evidence‐based, effective and empathetic SLT services in order to support individuals to live well despite these distressing symptoms. However, further in‐depth research using appropriate study designs, recruitment procedures, clinical applicability of research findings and standardised assessments with larger cohorts of patients will help researchers and clinicians alike to establish the true extent of these symptoms, and to ultimately aid patients in re‐establishing pre‐COVID‐19 health and wellbeing through engaging with evidence‐based intervention which is delivered by a comprehensive and skilled MDT.

## Author Contributions

**Kathleen McTiernan:** conceptualisation, methodology, writing original draft, review and editing, formal analysis. **Caoimhe Hughes:** methodology, formal analysis. **Órla Gilheaney:** writing – review and editing, methodology, formal analysis.

## Funding

The authors received no specific funding for this work.

## Ethics Statement

The authors have nothing to report.

## Conflicts of Interest

The authors declare no conflicts of interest.

## Data Availability

The authors have nothing to report.
